# Association analysis of telomere length related gene ACYP2 with the gastric cancer risk in the northwest Chinese Han population

**DOI:** 10.18632/oncotarget.16097

**Published:** 2017-03-10

**Authors:** Jianhui Li, Gang Ma, Xulong Zhu, Tianbo Jin, Jianxiong Wang, Cheng Li

**Affiliations:** ^1^ Department of Surgical Oncology, Shaanxi Provincial People's Hospital, Xi’an, Shaanxi 710068, China; ^2^ The Third Affiliated Hospital, the School of Medicine Xi’an Jiaotong University, Xi’an, Shaanxi 710068, China; ^3^ Key Laboratory of Resource Biology and Biotechnology in Western China (Northwest University), Ministry of Education, College of Life Sciences, Northwest University, Xi’an, Shaanxi 710069, China; ^4^ Xi’an Tiangen Precision Medical Institute, Xi’an, Shaanxi 710075, China

**Keywords:** gastric cancer (GC), single-nucleotide polymorphisms (SNPs), ACYP2, telomere length, association analysis

## Abstract

Gastric cancer (GC) is a complex multifactorial disease, and genetic factors are believed the predominant cause to the occurrence of GC. We sought to investigate the associations between single nucleotide polymorphisms (SNPs) in *ACYP2* gene and the risk of GC in the Northwest Chinese Han population. We recruited 302 GC cases and 300 controls from northwest China and selected 13 SNPs from *ACYP2* gene. SNPs were genotyped using Sequenom Mass-ARRAY technology. Odds ratios (ORs) and 95 % confidence intervals (CIs) were used to assess the association. Bonferroni's multiple adjustment was applied to the level of significance, which was set at *P* < 0.00078 (0.05/65). We found that the minor alleles of rs6713088, rs11125529, rs12615793, rs843711, rs11896604, rs843706 and rs17045754 significantly stimulated the risk of GC, and homozygous alleles of above SNPs except rs6713088 were also found increasing the GC risk (*P* < 0.05). Under additive model and recessive model, rs11125529, rs12615793, rs843711, rs11896604, and rs17045754 also activated the risk of GC (*P* < 0.05). However, after Bonferroni's multiple adjusted was applied to our data, no SNP in our study was significantly related to GC risk. Further results of haplotype analysis founds that the haplotypes “TTCTAATG” (rs1682111, rs843752, rs10439478, rs843645, rs11125529, rs12615793, rs843711, and rs11896604) and “AC” (rs843706 and rs17045754) were more frequency among patients with GC, on the contrary, the haplotypes “CG” had a protective role in the GC risk (*P* < 0.05). Our results indicate that *ACYP2* polymorphisms may influence the GC risk and may serve as a new precursory biomarker in the northwest Chinese Han population.

## INTRODUCTION

Gastric cancer (GC) is the second common cancer of gastrointestinal tumor worldwide with high mortality rate due to poor prognoses and inadequate therapy [1, 2], especially in China [3]. As it is well known, unhealthy lifestyles and unsafe surroundings are the main etiology of for the GC [4], furthermore, genetic factors are believed the predominant cause for the occurrence of GC, and some studies have verified that the variant of telomere length have an association with the risk of GC [5].

Telomere, a special structure, is composed of shorter repeated non-transcribed sequences (TTAGGG) and binding proteins. Telomere can not only protect the ends of chromosome against fusion and degradation but also plays an important role in cell apoptosis, cell transformation and immortalization. Some evidence indicated that variation of telomere length was related to increased several kinds of human cancer risk, including melanoma [6], breast cancer [7] and bladder cancer [8]. At the same time, study has reported that *ZNF308, TERC, TERT, OBFC1, NAF1, RTEL1, ACYP2* genes had a relationship with telomere length [9]. Furthermore, the variant of *ACYP2* gene was confirmed the association with coronary heart disease in Chinese Han population [10]. However, the association between *ACYP2* polymorphism and the GC risk has not been researched previously.

*ACPY2*, which is located in chromosome 2p16.2, encodes a small cytosolic enzyme acylphosphatase that can hydrolyze the synthetic of carboxyl phosophate bond [11]. Much of the information has displayed that acylphosphatase has an effect on the transport of Calcium ion across biological membranes and may be associated with cellular apoptosis [12, 13]. Previously *ACYP2* genetic polymorphism study was researched on single nucleotide polymorphisms ( SNPs ) associated with colorectal cancer risk in genome-wide association study [14]. However, if ACYP2 gene is also the shared risk gene for GC is unknown, and this need to be researched. It is curious to hypothesize that the polymorphisms of *ACYP2* may lead to different susceptibilities to GC. The purpose of the present case-control study was to identify the association between 13 high frequency SNPs of *ACYP2* and GC risk in the Northwest Chinese Han population.

## RESULTS

### Characteristic of the study participants GC

A number of 300 healthy controls and 302 GC patients were enrolled in our study. Baseline characteristics of the control and GC patients groups were evaluated, and we found that both gender and age have a significant difference between control group and case group (*P* < 0.001, *P* < 0.001 respectively) (Table 1). Therefore, the *ACYP2* polymorphisms analysis, along with age and gender, were adjusted further for unconditional multivariate regression analysis in this case control study.

**Table 1:** Characteristic of the control individuals and patients with gastric cancer

Characteristic	control (*N* = 300)	Case (*N* = 302)	*p*-value
Sex (%)			< 0.001^a^
female	120 (40%)	69 (22.8%)	
male	180 (60%)	233 (77.2%)	
Mean age ± SD	58.01 ± 5.14	60.42 ± 11.27	< 0.001^b^

^a^*P* value is calculated by Pearson's Chi-square test. ^b^*P* value is calculated by Welch's *t* test.

### SNPs associated with the GC risk under allelic model

The characteristic of candidate SNPs were exhibited in Table 2, containing position, role, risk allele and allele frequencies. Allele frequencies in cases and controls were tested for departure from Hardy-Weinberg equilibrium using a Fisher's exact test (Table 2). None was excluded because of having not a significant deviation from HWE (*P* > 0.05). Chi-square test was used to describe the association between the minor allele and the risk of GC. The odds ratio (OR), confidence interval (CI) and *p value* showed that the variants of seven SNPs had an association with the risk of GC in the allelic model analysis. From Table 2, the minor allele “G” of rs6713088 increased the risk of GC by 1.302-fold (95% CI = 1.034–1.64, *P* = 0.024) and the minor allele “G” of rs11896604 also associated with GC risk by 1.406-fold (95% CI = 1.06–1.865, *P* = 0.018). Similarly, rs11125529 minor allele “A”, rs12615793 minor allele “A” and rs843706 minor allele “A” elevated the GC susceptibility by 1.419-fold (95% CI = 1.066–1.889, *P* = 0.016), 1.392-fold (95% CI = 1.054–1.838, *P* = 0.02) and 1.347-fold (95% CI = 1.071–1.694, *P* = 0.011) respectively. Rs843711 risk allele “T’ frequency in case and control had a significant difference and the variant increased the GC risk (OR = 1.4, 95% CI = 1.115–1.758, *P* = 0.004). For rs17045754 risk allele “C”, it had the same effect on the risk of GC (OR = 1.376, 95% CI = 1.071–1.694, *P* = 0.011). The remaining sites were detached from the further model analysis. However, After Bonferroni's multiple adjustment, no variants of SNPs was significant associated with GC risk.

**Table 2:** Allele frequencies of candidate SNPs examined in the ACYP2 gene among the cases and controls and odds ratio estimates for gastric cancer

SNP ID	position	Role	Alleles A/B	MAF-case	MAF-control	HWE-P^a^	OR	95% CI	χ2	*P*^b^
rs6713088	54345469	Intron	G/C	0.440	0.377	0.623	1.302	1.034–1.640	5.059	0.024*
rs12621038	54391113	Intron	T/C	0.457	0.450	0.415	1.029	0.820–1.291	0.061	0.804
rs1682111	54427979	Intron	A/T	0.295	0.333	0.604	0.836	0.655–1.066	2.086	0.149
rs843752	54446587	Intron	G/T	0.270	0.248	0.877	1.119	0.864–1.448	0.727	0.394
rs10439478	54459450	Intron	C/A	0.439	0.425	0.237	1.058	0.842–1.329	0.233	0.630
rs843645	54474664	Downstream	G/T	0.258	0.237	0.749	1.123	0.864–1.460	0.755	0.385
rs11125529	54475866	Downstream	A/C	0.225	0.170	0.411	1.419	1.066–1.889	5.776	0.016*
rs12615793	54475914	Downstream	A/G	0.240	0.185	0.254	1.392	1.054–1.838	5.452	0.020*
rs843711	54479117	Downstream	T/C	0.498	0.415	0.287	1.400	1.115–1.758	8.426	0.004*
rs11896604	54479199	Downstream	G/C	0.232	0.177	1.000	1.406	1.060–1.865	5.625	0.018*
rs843706	54480369	UTR	A/C	0.493	0.419	0.341	1.347	1.071–1.694	6.517	0.011*
rs17045754	54496757	Intron	C/G	0.222	0.172	0.547	1.376	1.033–1.831	4.795	0.029*
rs843720	54510660	Intron	G/T	0.346	0.358	0.451	0.947	0.748–1.208	0.200	0.655

Abbreviations: SNPs: Single nucleotide polymorphisms; A: Miner alleles, B: Major alleles; MAF: Minor allele frequency;

HWE: Hardy-Weinberg equilibrium; OR: Odds ratio. CI: Confidence interval;

^a^*P* values were calculated using exact test; ^b^*P* values were calculated using Chi-square test; *P < 0.05 indicates statistical significance;

Bonferroni's multiple adjustment was applied to the level of significance, which was set at *P* < 0.00078 (0.05/65).

### Genotypes and the risk of GC

The genotype frequencies of SNPs in the GC patients and controls showed that, rs11125529, rs12615793, rs843711, rs11896604, rs843706 and rs17045754 were significantly correlated with GC risk by unconditional logistic regression adjusted by gender and age (*P* < 0.05) (Table 3). The rs11125529 mutant genotype “AA” compared to wild genotype “CC” and the rs12615793 mutant genotype “AA” compared to wild genotype “GG” were associated with a significant increased the risk of GC (rs11125529 OR = 4.008, 95% CI = 1.538–10.45, *P* = 0.005; rs12615793 OR = 3.68, 95% CI = 1.501–9.02, *P* = 0.004). The significantly risk effects were also found in the rs843711 variant genotypes “TT” and rs11896604 variant genotypes “GG” (rs843711 TT *vs* CC OR = 1.909, 95% CI = 1.17–3.115, *P* = 0.009, rs11896604 GG *vs* CC OR = 2.739, 95% CI = 1.197–6.265, *P* = 0.017). In addition, the rs843706 mutant genotype “AA” and rs17045754 mutant genotype “GG” were associated with a statistically significantly increased risk of GC compared with the “CC” genotype and “GG” genotypes respectively (rs843706 OR = 1.75, 95% CI = 1.067–2.872, *P* = 0.027; rs17045754 OR = 3.147, 95% CI = 1.252–7.906, *P* = 0.015). However, our data showed none of the SNPs had the association with GC susceptibility under the genotypes of heterozygous minor alleles (*P* > 0.05) (Table 3) and no difference of genotype distribution of rs6713088 was found between the cases and controls. Unfortunately, these SNPs neither in recessive model nor in addictive model were significantly associated with GC risk after Bonferroni's multiple adjusted.

**Table 3:** Distribution of genotypes of prominent SNPS and their associations with the risk of developing gastric cancer

SNP ID	Alleles A/B	genetype	No. (frequency)		Logistic regression	
			Case (%)	Control (%)	OR (95% CI)	*P*^a^
rs6713088	G/C	CC	97 (32.12)	114 (38)	1	
		GG	61 (20.20)	40 (13.33)	1.529(0.93–2.512)	0.094
		GC	144 (47.68)	146 (48.46)	1.144(0.793–1.65)	0.472
rs11125529	A/C	CC	186 (61.59)	204 (68)	1	
		AA	20 (6.62)	6 (2)	4.008(1.538–10.45)	0.005*
		AC	96 (31.79)	90 (30)	1.174(0.819–1.684)	0.382
rs12615793	A/G	GG	179 (59.27)	196 (65.33)	1	
		AA	22 (7.28)	7 (2.33)	3.68(1.501–9.02)	0.004*
		AG	101 (33.44)	97 (32.33)	1.183(0.829–1.688)	0.355
rs843711	T/C	CC	74 (24.5)	98 (32.67)	1	
		TT	73 (24.17)	47 (15.67)	1.909(1.17–3.115)	0.009*
		TC	155 (52.36)	155 (51.67)	1.281(0.87–1.887)	0.209
rs11896604	G/C	CC	183 (60.6)	203 (67.67)	1	
		GG	21 (6.95)	9 (3)	2.739(1.197–6.265)	0.017*
		GC	98 (32.45)	88 (29.33)	1.226(0.854–1.759)	0.269
rs843706	A/C	CC	72 (24.32)	96 (32.21)	1	
		AA	68 (22.97)	48 (16.11)	1.75(1.067–2.872)	0.027*
		AC	156 (52.7)	154 (51.68)	1.294(0.876–1.914)	0.196
rs17045754	C/G	GG	186 (61.59)	204 (68)	1	
		CC	18 (5.96)	7 (2.33)	3.147(1.252–7.906)	0.015*
		CG	98 (32.45)	89 (29.67)	1.224(0.854–1.754)	0.272

Abbreviations: A: Miner alleles, B: Major alleles; OR: Odds ratio, CI: Confidence interval;

^a^*P* values were calculated by Wald test adjusted by gender and age; *P < 0.05 indicates statistical significance.

Bonferroni's multiple adjustment was applied to the level of significance, which was set at *P* < 0.00078 (0.05/65).

We further analyzed the association between prominent SNPs and GC risk by Wald test using three models, containing dominant model, additive model and recessive model adjusted for gender and age (Table 4). We found that rs11125529, rs12615793, rs843711, rs11896604, and rs17045754 were related to an increased risk with GC under both additive model and recessive model (*P* < 0.05). For the rs843706, under additive model, the association with GC risk was obvious (OR = 1.32, 95% CI = 1.033–1.687, *P* = 0.027), while, the association was not exist under recessive model. None of SNPs have a connection with the risk of GC under dominant model.

**Table 4:** Association of prominent SNPs with the gastric cancer based on logistic tests adjusted by gender and age

SNP ID	Minor allele	Dominant model		Additive model		Recessive model	
		OR (95% CI)	*p*^a^	OR (95% CI)	*p*^a^	OR (95% CI)	*p*^a^
rs6713088	G	1.231 (0.87–1.74)	0.241	1.218 (0.959–1.547)	0.106	1.413 (0.902–2.213)	0.131
rs11125529	A	1.344 (0.951–1.899)	0.094	1.438 (1.073–1.928)	0.015*	3.804 (1.47–9.845)	0.006*
rs12615793	A	1.348 (0.9586–1.897)	0.086	1.436 (1.078–1.913)	0.013*	3.47 (1.428–8.429)	0.006*
rs843711	T	1.429 (0.989–2.064)	0.058	1.372 (1.076–1.748)	0.011*	1.625 (1.068–2.472)	0.023*
rs11896604	G	1.363 (0.9656–1.923)	0.078	1.398 (1.051–1.86)	0.021*	2.564 (1.13–5.815)	0.024*
rs843706	A	1.404 (0.9669–2.037)	0.075	1.32 (1.033–1.687)	0.027*	1.478 (0.9678–2.256)	0.071
rs17045754	C	1.357 (0.96–1.918)	0.084	1.411 (1.051–1.894)	0.022*	2.946 (1.182–7.343)	0.02*

Abbreviation: SNP: Single nucleotide polymorphisms;

^a^*P* values were calculated by Wald test adjusted by gender and age; *P < 0.05 indicates statistical significance;

Bonferroni's multiple adjustment was applied to the level of significance, which was set at *P* < 0.00078 (0.05/65).

### Haplotypes and the risk of GC

**Figure 1:**
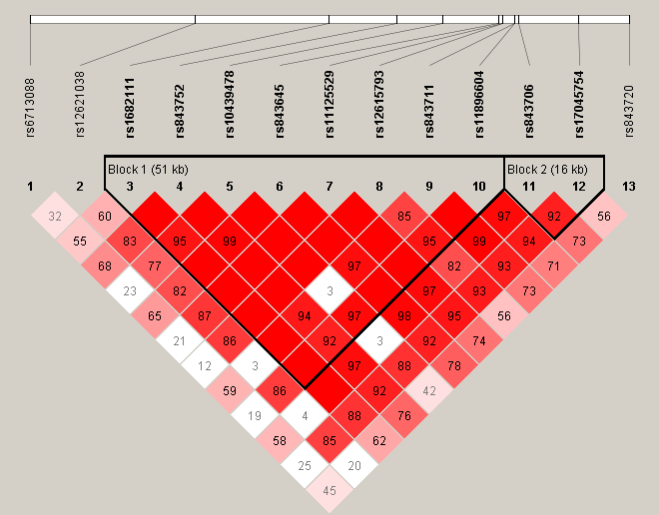
Haplotype block map for part of the SNPs in *ACYP2* gene Linkage disequilibrium plots containing thirteen SNPs from 2p16.2. Standard color frame is used to show LD pattern. Two blocks in the figure showed higher LD. Red squares display statistically significant associations between a pair of SNPs, as measured by D’; darker shades of red indicate higher D’.

Using SHEsis software, two blocks were detected in studied *ACYP2* SNPs by haplotype analyses (Figure 1), In block 1, a pair of eight SNPs had an linkage disequilibrium: rs1682111, rs843752, rs10439478, rs843645, rs11125529, rs12615793, rs843711, and rs11896604 and block 2 was containing two SNPs: rs843706 and rs17045754. Haplotypes with frequency more than 1% in the present study were listed in Table 5. The analysis result showed that haplotype “TTCTAATG” had a significant connection with the risk of GC after the adjustment by age and gender (OR = 1.453, 95% CI = 1.083–1.951, *P* < 0.013). At same time, the rs843706 minor allele “A” and rs17045754 minor allele “C” constituted the haplotype “AC” also increased the risk of GC (OR = 1.444, 95% CI = 1.072–1.944, *P* = 0.016). On the contrary, the haplotype “CG” was associated with a 0.773-fold reduced risk of GC (95% CI = 0.605–0.989, *P* = 0.041) (Table 5). The other haplotypes were exhibited to be irrelevant to GC risk.

**Table 5:** *ACYP2* haplotype frequencies and the association with gastric cancer among the cases and controls

SNPs	Haplotype	Freq (case)	Freq (control)	*P*^a^	OR (95% CI)	*P*^b^
rs1682111|rs843752|rs10439478|rs843645|rs11125529|rs12615793|rs843711 |rs11896604	ATATCGCC	0.293	0.328	0.192	0.8139 (0.6316–1.049)	0.111
	TTCTAATG	0.227	0.170	0.014*	1.453 (1.083–1.951)	0.013*
	TGAGCGTC	0.248	0.231	0.474	1.042 (0.791–1.373)	0.771
	TTCTCACC	0.013	0.015	0.791	1.03 (0.379–2.801)	0.954
	TTCTCGCC	0.182	0.227	0.051	0.8224 (0.607–1.115)	0.208
rs843706|rs17045754	AC	0.220	0.166	0.019*	1.444 (1.072–1.944)	0.016*
	AG	0.274	0.253	0.427	1.031 (0.788–1.35)	0.822
	CG	0.507	0.576	0.017*	0.7734 (0.605–0.989)	0.041*

^a^*P* values were calculated by two side Chi-square test; ^b^*P* values were calculated by Wald test adjusted by gender and age; *P < 0.05 indicates statistical significance.

## DISCUSSION

In our study, we reported the association of tag 13 SNPs in *ACYP2* gene with the risk of developing GC in a Northwest Chinese population. Our results reflected that *ACYP2* rs6713088, rs11125529, rs12615793, rs843711, rs11896604, rs843706 and rs17045754 were associated with an increased GC risk in the study population; and the associations were disappeared after Bonferroni's multiple adjusted. Furthermore, Haplotype analysis results showed that haplotypes “TTCTAATG” and “AC” were associated with increased GC risk by unconditional logistic regression analysis adjusted by gender and age. Because of Bonferroni correction is a more rigorous correction method, and truly significant differences may be considered non-significant as a result of type II errors in some cases. Furthermore, we found some SNPs have an association with the risk of GC at the extremely significant difference level *P* < 0.01, such as minor allele “A” of rs11125529, minor allele of “A” of rs12615793 and minor allele “T” of rs843711. Then *ACYP2* gene may have a close connection with the risk of GC. Despite the mechanism of the association between *ACYP2* gene and the GC is unclear.

We found a possible association between *ACYP2* gene and the risk of gastric cancer by reviewing the literatures. Many researches indicated that *ACYP2* gene had a connection with the telomere length, and rs11125529 was published associate with shorter telomere length [15]. Telomeres, the protein-bound DNA repeat structures, are the extreme ends of chromosomes that play a vital role in maintaining genomic stability [16, 17]. It is believed that those individuals who have the shorter telomeres might be more sensitive to some certain diseases compared to the controls. [18]. For example, rs7675998 was found to be related to the onset age of coronary heart disease in a case-control study [10]. Study had reported that gastric patients had remarkably shorter average telomere length than matched controls and the shorter average telomere length had a significantly increased GC risk in the Chinese Han population [5]; in addition, this result also found in the high-risk Polish population [19]. Our result showed that rs11125529 A < C had the association with the increased risk of GC. Thus, *ACYP2* genetic variants determining telomere length may have an effect on the GC risk.

Presently, some articles also found that polymorphism of ACYP2 gene have related to cancers. For example, rs1682111 and rs17045754 were associated with the recurrence of breast cancer [20], and three SNP rs1682111, rs11896604 and rs843720 associated with lung cancer [21]. Additional, the rs11896604 significantly decreased the risk of high altitude pulmonary edema after Bonferroni correction [22], and rs843706 and rs17045754 were found increase the risk of ischemic stroke [23]. In this study, our result indicated variants of ACYP2 may be associated with activate the risk of GC. These studies indicate that variants of ACYP2 may influence the risk of many kinds of diseases, and these studies containing this case control study only found the association with diseases in Chinese Han population. Subsequent studies need to be conducted on our findings.

In our study, there were still some deficiencies, for example the relatively small sample size which could decrease the statistical power to detect the function of SNPs on risk of GC. Furthermore, the population of our study is limited in Chinese Han population, and the associations we reported have not been researched in other populations. Lastly, some indicators associated with GC were not compared such as drinking and *Helicobacter pylori* infection and so on, because of lack of the detailed clinical information which was very difficult to collect. Then, in future study, we need to collect enough information on the characteristic of study subjects in the sample recruiting process.

In summary, our case-control study explored the correlation between 13 SNPs of telomere length related gene ACYP2 with the risk of GC, and the results indicated *ACYP2* gene may associated with GC in the Chinese Han population from northwest. This research provided a theoretical evidence and selection for the early diagnosis of GC, and ACYP2 may serve as a potentially precursory biomarker among Chinese population. Whether the results of the study are applicable to other races is still required confirmation by a further research in a larger cohort of GC patients of other nations. Moreover, the details need to be researched to explain the mechanism of the *ACYP2* gene associated with GC risk.

## MATERIALS AND METHODS

### Subject recruitment

The cases were recruited between May 2013 and May 2016 from Shaanxi Provincial People's Hospital. Eligible cases were 302 patients who were diagnosed by two experienced pathologists, and confirmed gastric cancer according to the World Health Organization criteria [24]. Patients with the following situations were excluded, containing inflammation autoimmune disorders, family history of cancer, and accepted radiotherapy and chemotherapy. The healthy controls were 300 individuals who were cancer-free randomly selected from the hospital in the same study period. All of the information of healthy controls interviewed by the professional interviewers, containing age, gender, family history of cancer and occupational exposure to carcinogens, and the person who possessed these unhealthy factors were removed from this study.

### Ethics committee statement

The use of human tissue and the protocol in this study were strictly conformed to the principles expressed in the Declaration of Helsinki and were approved by the Ethical Committee of Shaanxi Provincial People's Hospital, Xi’an Jiaotong University and Northwest University for approval of research involving human subjects. The individual in this manuscript has given written informed consent to publish these case details.

### SNP selection and genotyping

Peripheral blood samples were collected in an anticoagulation tube and stored at –80°C until detection before subjects had received other therapies. Depend on manufacturer′s instructions of the GoldMag-Mini Purification Kit (GoldMag Co.Ltd. Xi′an city, China), genomic DNA was isolated from blood leukocytes samples. At the same time, the concentrations and purity of the DNA were measured by using the NanoDrop 2000 (Thermo Fisher Scientific, Waltham, Massachusetts, USA) at a wavelength of A260 and A280 nm.

A total fourteen SNPs were selected at a minor allele frequency > 5% in the HapMap Chinese Han Beijing (CHB) population. Sequenom Mass-ARRAY RS1000 (Sequenom, San Diego, CA) was used to genotype the SNPs rs6713088, rs12621038, rs1682111, rs843752, rs10439478, rs843645, rs11125529, rs12615793, rs843711, rs11896604, rs843706, rs17045754, and rs843720. Data management and analysis were managed by using Sequenom Typer 4.0 Software (Sequenom Co. Ltd) [25, 26]. The PCR primers for the fourteen selected tSNPs were shown in Table 6.

**Table 6:** Primers used for this study

SNP-ID	1st-PCRP	2nd-PCRP	UEP_SEQ
rs6713088	ACGTTGGATGACACACACAGACTCCTTCAC	ACGTTGGATGGTCACCAAAACACGTAATG	gaggcCAGAATGGTCCACTAGAGA
rs12621038	ACGTTGGATGATTGTGCTAGGCACTTTAGG	ACGTTGGATGGGCATAAGTTTTATTGCCTC	ccATTGCCTCAGCTAGACT
rs1682111	ACGTTGGATGGAATTGCTGGGTTATTTGGC	ACGTTGGATGGCCAGTGGGAATGCAAAATG	tgtcATGCAAAATGAAACAGACACTT
rs843752	ACGTTGGATGTCCTCTTTTCAGAAACCTGC	ACGTTGGATGGAGACAACATAATGGAGGTC	cGAGTTTGGGTTTGAGGT
rs10439478	ACGTTGGATGTAGCACAAGACCTACACTGG	ACGTTGGATGCTACACTCTCCAGAGGAATG	TTGCTGTTTTCCCAGAA
rs843645	ACGTTGGATGGAAATCTGAATACCACCTAC	ACGTTGGATGACAGTGCCTTTAGCAAGGTG	TCATAGGCACTACTGTATC
rs11125529	ACGTTGGATGGAGCTTAGTTGTTTACAGATG	ACGTTGGATGCCGAAGAAAAGAAGATGAC	AGAAAAGAAGATGACTAAAACAT
rs12615793	ACGTTGGATGTTTGAGCTTAGTTGTTTAC	ACGTTGGATGATCTTGGCCCTTGAAGAA	AAATTGAGTGACAAATATAAACTAC
rs843711	ACGTTGGATGGACAAAGGACCTTACAACTC	ACGTTGGATGTGCCTTGTGGGAATTAGAGC	gggaTCAGGGAACCAGTGCAAA
rs11896604	ACGTTGGATGAAGTCAGAATAGTGCTTAC	ACGTTGGATGTGTCTCTGACCTAGCATGTA	GTTAAGCTTGCAAGGAG
rs843706	ACGTTGGATGTGAAAGCCATAAATATTTTG	ACGTTGGATGTGAATAACTTGGTCTTATC	cACTTGGTCTTATCTGATGC
rs17045754	ACGTTGGATGCTGTAAAAGTTCTGGCATGG	ACGTTGGATGGAAATCAGGGATATTAGTGC	caggTATTCAGCTTCCTAGAGTTA
rs843720	ACGTTGGATGCTTCACAACACTCCTGTAAG	ACGTTGGATGAGTCAGAGCTAGACCTCTGG	ccccAATCTGTCTCAGGGTCTT

### Statistical analysis

Microsoft Excel and SPSS 16.0 (SPSS, Chicago, IL, USA) were used to perform statistical analyses. Throughout the document, *P values* were two-sided, furthermore, *P* < 0.05 was thought to have a statistical significant. The differences in the characteristics of the case and control study populations were compared using the chi-squared test (for gender variables) and Welch's *t* tests (for age variables). The exact test was used to determine whether the tSNPs departure from Hardy–Weinberg equilibrium (HWE), and Chi-squared test/Fisher's exact test was used to calculate the differences in frequency distributions of alleles between cases and controls [27]. In order to assess the association between each genotype and the risk of GC, three models were used, including dominant model, recessive model, and additive model. Bonferroni correction was using in our date to eliminate the probability of false-positive finding, and the level of significance was set at *P* < 0.00078 (0.05/65).

Finally, in the LD analysis, the patterns of linkage disequilibrium (LD) and haplotypes construction were evaluated by the the SHEsis software platform (http://analysis.bio-x.cn/myAnalysis.php) and Haploview software package (version 4.2) [28]. Control samples were used to the haplotype construction. The linkage disequilibrium degree of the two SNPs is measured by D’ value and D’ confidence interval is used to divide haplotype block. The D’ value is between 0 and 1. The closer the |D’| value is to 1, the higher the level of linkage disequilibrium was between the loci. Unconditional logistic regression model adjusted for age and gender was used to calculate odds ratios (ORs) and 95 % confidence intervals (95 % CI) for each polymorphism [29].

## Abbreviations

GC: Gastric Cancer
SNP: Single nucleotide polymorphism
OR: Odds ratio
CI: Confidence interval
LD: Linkage disequilibrium
